# Behavior of glioblastoma brain tumor stem cells following a suborbital rocket flight: reaching the “edge” of outer space

**DOI:** 10.1038/s41526-023-00341-9

**Published:** 2023-12-18

**Authors:** Cesar A. Garcia, Paola Suárez-Meade, Mieu Brooks, Adip G. Bhargav, Michelle L. Freeman, Lawrence M. Harvey, John Quinn, Alfredo Quiñones-Hinojosa

**Affiliations:** 1 Department of Neurosurgery, Mayo Clinic, Jacksonville, FL USA; 2 Stanford University School of Medicine, Palo Alto, CA USA; 3 Department of Neurological Surgery, University of Kansas Medical Center, Kansas City, KS USA; 4 Department of Critical Care Medicine, Mayo Clinic, Jacksonville, FL USA; 5 Center for Applied Space Technology, Cape Canaveral, FL USA; 6 EXOS Aerospace Systems and Technologies, Greenville, TX USA

**Keywords:** CNS cancer, Cancer stem cells

## Abstract

The emerging arena of space exploration has created opportunities to study cancer cell biology in the environments of microgravity and hypergravity. Studying cellular behavior in altered gravity conditions has allowed researchers to make observations of cell function that would otherwise remain unnoticed. The patient-derived QNS108 brain tumor initiating cell line (BTIC), isolated from glioblastoma (GBM) tissue, was launched on a suborbital, parabolic rocket flight conducted by EXOS Aerospace Systems & Technologies. All biologicals and appropriate ground controls were secured post-launch and transported back to our research facility. Cells from the rocket-flight and ground-based controls were isolated from the culture containers and expanded on adherent flasks for two weeks. In vitro migration, proliferation, and stemness assays were performed. Following cell expansion, male nude mice were intracranially injected with either ground-control (GC) or rocket-flight (RF) exposed cells to assess tumorigenic capacity (*n* = 5 per group). Patient-derived QNS108 BTICs exposed to RF displayed more aggressive tumor growth than the GC cells in vitro and in vivo. RF cells showed significantly higher migration (*p* < 0.000**0**) and stemness profiles (*p* < 0.01) when compared to GC cells. Further, RF cells, when implanted in vivo in the brain of rodents had larger tumor-associated cystic growth areas (*p* = 0.00029) and decreased survival (*p* = 0.0172) as compared to those animals that had GC cells implanted.

## Introduction

Space exploration has expanded rapidly since the beginning of the 21st century. Technological advancements have allowed us to create recoverable rockets or reusable launch vehicles (RLV) that can be reused multiple times within a narrow launch window^1,2^. Space travel poses several risks, such as exposure to microgravity, hypergravity, and ionizing radiation^3,4^. The question of how such exposures may affect human physiology is relevant as we explore the possibility of extended space travel. Earlier studies have demonstrated how microgravity on the International Space Station (ISS) can affect cancer cell biology by altering mechanisms such as apoptosis, proliferation, and migration^5–7^. These same studies demonstrated that altered gravity conditions could push thyroid and breast cancer cells to adopt either pro- or anti-tumorigenic behaviors^5–7^. Little is known how rocket flight or space travel may affect patient-derived GBM tumor cells.

GBM is the most common malignant primary tumor of the central nervous system, characterized by poor clinical outcomes. With treatment, the median survival rate for GBM ranges from 6–14 months, and the 5-year survival is 6.8%^8–11^. A subpopulation of cells within GBM are referred to as brain tumor stem cells or brain tumor initiating cells (BTICs), a stem-like population of cells that have been shown to be invasive, resistant to chemotherapy and radiation treatment, and are the likely culprits for tumor recurrence after standard therapy has been applied^12–14^.

With regards to brain cancer, simulated microgravity research has shown that microgravity reduces the malignant behaviors of GBM tumor cells by increasing apoptosis and sensitizes them to treatment^5,15,16^. In the present study, we explored how rocket flight could influence the tumorigenic capacity of BTICs isolated from a tumor sample collected from a male patient who underwent surgical resection for GBM. The patient-derived BTICs were sent to the edge of space on a rocket flight expedition, where they were exposed to high altitude, altered gravity conditions, and ionizing radiation. In this study we report the behavior of these BTICs after the exposure to the environment of rocket flight.

## Results

### BTICs were exposed to altered gravity during rocket flight

**Fig. 1:**
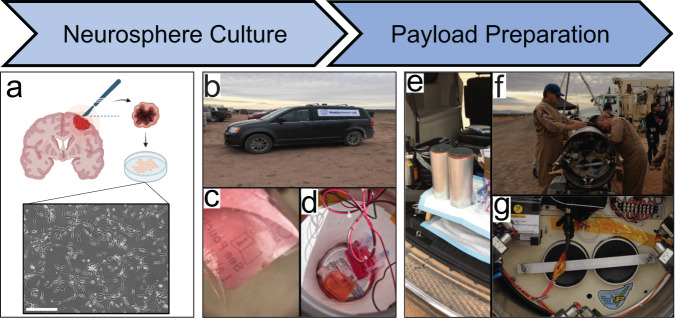
Brain tumor initiating cell (BTIC) neurospheres were prepared for a suborbital rocket-launch. **a–g** QNS108 neurospheres were cultured in 7 ml Origen bags, placed in biological research in a cannister (BRIC) containers, inserted into the rocket payload module and prepared for rocket-launch. Scale bar = 50 µm. Figure 1a was created with Biorender.com. Personnel from Exos aerospace company provided permission for use of all images.

**Fig. 2:**
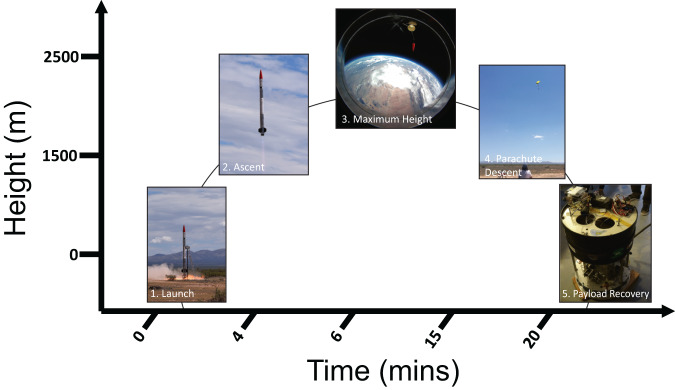
Rocket Flight. The rocket with a secured biological payload followed a parabolic flight path with a post-launch recovery of biological samples.

**Fig. 3:**
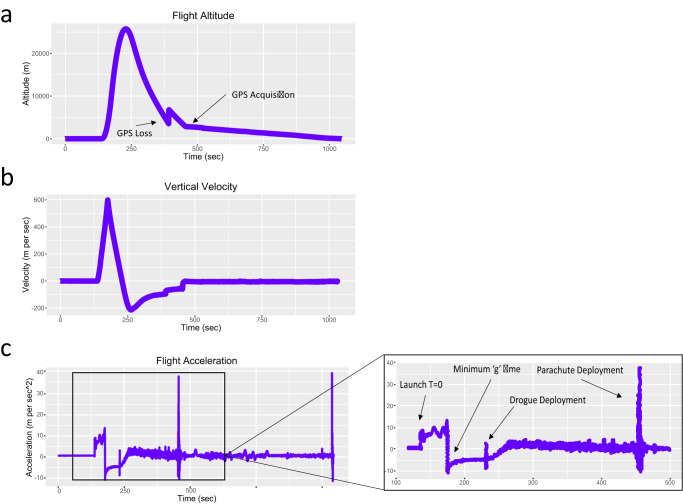
Brain tumor initiating cells experienced vertical altitude, velocity, and acceleration changes throughout the course of a suborbital rocket-launch. **a** Altitude, **b** vertical velocity, and **c** acceleration were recorded with flight telemetry.

The QNS108 cells were placed in 7 ml Origen Cell Culture bags and transported by road from Jacksonville, FL to Spaceport America in Las Cruces, New Mexico over 48-h, while maintained in a portable incubator set at 37 °C (Fig. 1a–c). A GC culture bag was kept in the portable incubator while a RF culture bag was placed in a biological research in a cannister (BRIC) container that served as the rocket payload (Fig. 1d). The BRIC container was integrated onto the payload of the Suborbital Autonomous Rocket with GuidancE (SARGE) vehicle (Exos Aerospace Systems & Technologies Inc., Greenville, TX). The SARGE vehicle employs a LOX-ethanol propulsion module based on the LE23000FC series engines, producing approximately 5500 lbs of thrust (Fig. 1e–g). The rocket was launched at Spaceport America where it followed a parabolic flight path (Fig. 2). The rocket returned via automatic guiding system to land near the launch site for rapid retrieval of payloads. Altitude, vertical velocity, and acceleration were recorded over the course of the rocket flight with onboard telemetry readings (Fig. 3). Over the course of rocket flight, a max altitude of 25.74 km (84,448 ft), a max velocity of 598.60 m per s, and a max acceleration of 13.32 m per s^2^ were recorded over a total time course of 16 minutes from launch to landing (Fig. 3a–c).

The time duration in hypergravity was approximately 39 seconds at 1.5 g’s experienced during take-off, indicated by the increasing positive acceleration at the beginning of the flight (Fig. 3c). Immediately following the peak of positive acceleration during rocket ascent, the rocket reached the max altitude of the parabolic flight path and experienced microgravity or minimum ‘g’ time with a recorded acceleration measured at −11.06 m per sec^2^ at a time point of 176.38 seconds (2.9 minutes) (Fig. 3c). A parachute was released as the rocket descended and was guided to land close to the launchpad based on GPS navigation and orientation. Biological samples were recovered from the payload container following the landing and returned to the portable incubator.

### RF exposed cells experienced phenotypic changes

**Fig. 4:**
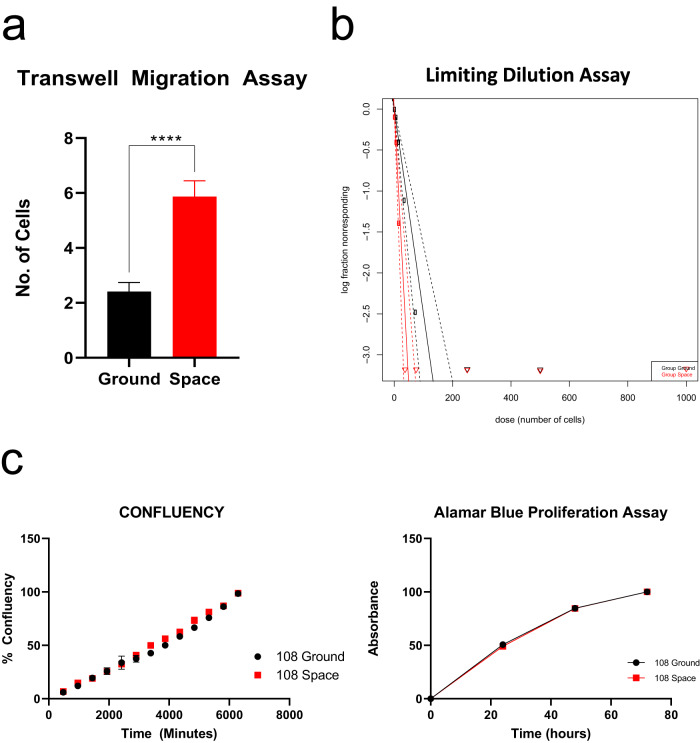
BTICs display increased migration in vitro after a suborbital rocket-launch but maintain the same proliferative capacity as ground controls. **a** Transwell migration assay was performed to evaluate the migratory ability of RC and GF BTICs. RF BTICs demonstrate a significantly higher migratory profile when compared to GC cells (****p < 0.0001). Scale bar = 3 mm. b) Limiting dilution assay demonstrated higher stemness in cells after suborbital rocket launch. Confidence intervals for 1/(stem cell frequency) were 27.2–60.8 and 10.5–22.6 in GC and RF groups, respectively. The overall test for differences in stem cell frequencies between groups was significant (**p < 0.01). **c** Proliferation assays using the CellCyte X technology and the Alamar Blue techniques did not show any differences between groups. All error bars represent standard error of the mean (SEM).

RF and GC BTICs (QNS108) were brought back to the laboratory 24-hours post-flight and were expanded in vitro and their invasive and stemness characteristics were evaluated. QNS108 RF cells presented a significantly higher migratory profile (*p* < 0.0001, Fig. 4a), as well as higher stemness properties (*p* < 0.001, Fig. 4b) when compared to GC. To further characterize the behavior of RF cells, we performed an Alamar blue assay and measured the confluency percentage with CellCyte technology to evaluate cellular proliferation. Interestingly, we did not find any significant differences in proliferation between groups (Fig. 4c).

### RF-exposed BTICs decreased survival in vivo

**Fig. 5:**
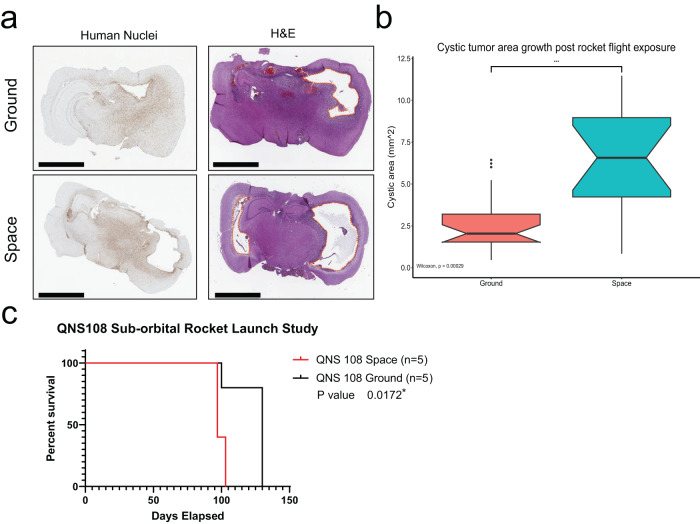
BTICs display larger cystic tumor growth in vivo and decrease survival after a suborbital rocket-launch. **a** Human nuclei (HuNu) and H&E staining BTICs, (QNS108) demonstrate whole-brain invasion accompanied with cystic lesions for GC and RF groups following rocket flight. **b** All tumor-associated cystic lesion areas on H&E stains were measured when observed on collected brain slices. Mice implanted with RF BTICs grew larger cystic areas as represented with boxplots, (*p < 0.05, **p < 0.01, ***p < 0.001, ****p < 0.0001). **c** Male-derived BTICs (QNS108) experienced ro**c**ket flight and were intracranially injected into nude mice postflight. RF BTICs caused decreased survival when compared to GC cells. (*p < 0.05, **p < 0.01, ***p < 0.001, ****p < 0.0001).

RF and GC-exposed cells were implanted into groups of 5 male nude mice, where tumor growth and survival were compared between the two cellular populations. QNS108 tumor growth was characterized by invasive spread across both hemispheres with the formation of cystic lesions for both groups, which has been observed previously^13^. The cystic lesion area accompanying tumor growth was measured for H&E stained slides. Larger cystic lesions formed in murine brains implanted with RF cells with a median area of 6.57 mm^2^ (4.23 mm^2^ (first quartile) –8.98 mm^2^ (third quartile)) vs the GC cells with a median area of 2.04 mm^2^ (1.53 mm^2^ (first quartile) –3.20 mm^2^ (third quartile)), (*p* = 0.00029; Fig. 5a, b).

It was observed that the RF group had decreased survival with a median survival time of 97 days compared to the GC group with a median survival of 130 days (*p* = 0.0172; Fig. 5c). The survival study indicates that the brief exposure to altered gravity conditions induced a permanent or long-lasting change within the cells which translated into a more aggressive phenotype. The exact transformation remains unknown but poses an active area of inquiry for our team and others.

## Discussion

The advent of space medicine has afforded opportunities to explore biological processes in unique environments like microgravity. Recent studies of cell biology performed on the International Space Station (ISS) have shown how biological behaviors can be influenced by altered gravity conditions^7,17–20^. For example, cell behaviors controlling proliferation, migration, and apoptosis were altered aboard the ISS or on rocket flight. For example, two studies were performed on the TEXUS 54 rocket mission conducted by the Swedish space agency where ovarian and thyroid cancer cell cytoskeletal dynamics were studied over the course of a parabolic rocket flight^20,21^. Over the two studies, the researchers demonstrated how hypergravity and microgravity altered actin and focal adhesion complex expression based on immunocytochemistry and real-time microscopy. Cells were shown to sense gravity conditions through cytoskeletal dynamics, which are known to transduce biomechanical signals to biochemical signals that can alter cellular behavior, although the signal transduction mechanisms are poorly understood. In our study, the BTICs likely experienced altered cytoskeletal changes in response to altered gravity conditions, which may present one mechanism for how cells transitioned to more tumorigenic phenotypes noted in later follow-up experiments.

Environments such as microgravity are difficult to replicate in normal earth-based settings and attempts have been made with machines such as rotating wall vessels or clinostats^22,23^. However, re-creating the environment of space travel or rocket flight is difficult. Space travel provides a unique environmental research laboratory to query biological phenomenon.

With that purpose, we isolated primary GBM tumor cells from a patient and transported them to the edge of space in a sub-orbital rocket-launch. Upon return, we tested the cellular behavior of cells and observed increased migration and invasion profiles, with no differences in cell proliferation. Further, we orthotopically transplanted the cells into mice and observed an increase in cystic lesion tumor formation and lower survival rate when compared to GC controls. The course of rocket flight would have exposed the cells to hypergravity, microgravity, and earth’s gravity throughout the duration of a parabolic flight path and parachute-descent. In addition, the cells were exposed to increased ionizing radiation in the upper atmosphere, although the exposure was not measured. Extra stress experienced by both groups of cells during cross-country transport may have included hypoxic and altered temperature conditions, as the portable incubator did not have a gas regulator nor did the launch area have a shelter. Additionally, neither the BRIC nor the payload compartment had temperature control. Hypoxia is a common feature of the tumor microenvironment and has been shown to maintain the glioma stem cell niche^24–26^. BTICs are cells that belong to the stem cell niche and their ability to proliferate, promote VEGF-mediated angiogenesis, tumor recurrence, and survive within the hypoxic environment are tied to their ability to express Hypoxia-inducible factor (HIF)-1 alpha and HIF-2 alpha in response to hypoxic conditions^24–26^. Overall, the RF cells experienced several added environmental stressors, one of which may have included increased hypoxia, enrichment of the cancer stem cell population, and upregulation of pro-tumorigenic behaviors in vitro and in vivo.

Our findings suggest that BTICs transformed to adopt a more tumorigenic phenotype after rocket-flight exposure. Applying stress to a population of cells can create a selective pressure, where resistant populations survive while sensitive populations die off. In our hands, the surviving cell populations of the RF group had greater invasive capacity that was acquired after exposure to extreme environmental conditions. Furthermore, we were able to observe a decreased survival in vivo and increased migration in vitro, suggesting a more aggressive phenotype of the BTIC (QNS108) line following RF. A study by Shi Z. et al., in which microgravity was modeled in vitro showed the opposite results on GBM aggressiveness^5^. Although other groups have demonstrated decreased proliferation in GBM cells subjected to microgravity conditions, here we did not find any differences in BTIC proliferation between RF and GC groups^26^.

In a clinical context, it has been shown that surviving cell populations within a tumor microenvironment following a course of chemo/radiation therapy are often resistant to treatment and serve as the seeds for later recurrence^11,13,27,28^. The enriched or selected pool of cells following rocket-flight appear to have retained their changed properties for a two-week growth period prior to being implanted in mice and decreasing survival. The finding suggests that even brief exposure to altered gravity conditions can influence overall cell functions or select for more aggressive clonal populations which persist over time. As was observed in our cell line, persisting human physiological changes following space flight were explored in the NASA Twins study^4^. Here, one identical twin sibling flew and remained on the ISS for one year, while the second sibling remained on Earth. Exposure to spaceflight-associated factors such as microgravity, cosmic radiation, and high-performance stress largely led to transient changes in areas like gene expression, cognition, immune cell function, and muscle mass with most metrics returning to baseline once the astronaut returned to Earth. However, some persistent biological changes that were present up to six months post-space flight included increased DNA damage/instability, telomere loss/shortening, increased chromosomal inversion frequency, and cognitive decline^4^. Hence, although most physiological changes were transient and only observed during space-travel, some permanent changes did occur and were largely seen at the genetic and DNA level. In our case, we compared two pools of cells collected from the same patient and same starting population and found that a few minutes of rocket flight could influence overall tumor formation up to two weeks later. What remains unknown are the genetic or molecular drivers of the changes observed in the rocket-flight exposed population.

Further areas of inquiry include how sex differences may affect physiological responses to the stress of space travel. Our study included a male-derived cell line implanted in male mice, but as has been shown by us and others, biological sex can affect tumor growth and clinical outcomes by operating at a molecular level^13,29^. A proposal for a future experimental design would be to track the phenotypic and genetic evolution of paired male and female cell lines when cultured in a microgravity setting aboard the ISS or on a repeat sub-orbital rocket flight.

As space exploration begins to expand, two major goals can be achieved. One is to better understand if and how space travel affects human physiology. Second is to use space as a laboratory to explore biological questions and generate solutions for existing health problems we experience back on Earth, like cancer. The questions of how biology changes in microgravity are crucial to understand as we attempt to conduct long-term space expeditions that will take us to Mars and beyond.

## Methods

### Cell Culture

All patient samples were collected following approval by the Mayo Clinic, Jacksonville FL institutional review board (IRB), informed written patient consent, and in accordance with the U.S. common rule. The male patient-derived QNS108 brain tumour-initiating cells (BTICS) were grown as reported previously^13,14^. In brief, cells were cultured on adherent flasks coated with laminin under normoxic conditions (37 °C, 5% CO_2_, 20% O_2_). Cell culture media used was DMEM/F12 (Gibco™) supplemented with 1% Anti-anti, 2% Gem21 NeuroPlex™, FGF, and EGF. Media was changed every 2–3 days.

### Transportation to rocket

Prior to departure, BTICs (QNS108) were passaged into two 7 ml Origen cell culture bags where they grew in suspension as neurospheres (Fig. 1a). One culture bag was designated as a ground control (GC) group and the second was designated as the rocket flight (RF) group. Both cell culture bags were placed in a portable incubator maintained at 37 °C and transported by ground over 48-hrs from Jacksonville, FL to Spaceport America in Las Cruces, New Mexico (Fig. 1b). The van was transformed into a portable laboratory where the payload was prepared. Cells in culture bags and biological materials were secured inside a hollow aluminum cylinder called a BRIC – “biological research in a cannister,” developed by NASA for research in microgravity (Fig. 1c, d). The BRIC containers have been designed to house plants, petri dishes, bacteria, cells, and biological materials and can be equipped with gas-controlled and temperature-regulated chambers and automated fixation or media changing systems^30–32^. The BRIC container used for our experiment did not have an oxygen/gas-controlled chamber or temperature regulator. Therefore, those parameters of gas control and temperature were unregulated after payload integration and until retrieval post-flight. Biorender.com was used to create part of Fig. 1a.

### Rocket-launch

One cell culture bag was packaged inside the BRIC container and the second culture bag was kept as a ground control. The BRIC was sealed and integrated into the payload module of the SARGE II rocket designed by EXOS Aerospace Systems & Technologies (Fig. 1e–g). The payload was launched as part of a suborbital reusable launch vehicle (SRLV) test launch (Fig. 2). A parabolic rocket-flight was conducted to generate forces of micro- and hypergravity. After launch, the rocket was recovered via parachute descent and biological materials were removed from the payload module and returned to the portable incubator. Samples were transported back to our research laboratory post-flight by ground transportation. Vertical acceleration, velocity, height, and G-forces were measured with onboard real-time telemetry (Fig. 3). Exposure to ionizing radiation was not recorded.

### Transwell migration assay

The migration capacity of BTICs (QNS108) was measured via the transwell migration assay as previously described^13^. Briefly, CoStar 6.5 mm inserts containing 8.0μm pore polycarbonate membrane were utilized. A total of 4 × 10^4^ GBM cells were seeded in the top chamber of the insert with an FBS gradient of 2% between the top and bottom chambers used as chemoattractant for migration. Cells were incubated at 37 °C in 5% CO_2_ for a period of 12 hours in four replicates. After incubation, cells were fixed with 4% PFA and the upper side of the insert was swabbed to remove cells that did not migrate through the membrane. Migrated cells attached to the opposite of the membrane were permeabilized using 0.1% triton solution in PBS and then stained with DAPI (1:1000 dilution). Membranes were imaged with a Zeiss LSM800 Confocal Microscopes, obtaining 9 random fields per membrane. The number of migrating cells were quantified with Fiji, ImageJ, software. These experiments were performed in triplicate.

### Limiting dilution assay

To estimate the brain tumor-initiating cell frequency in a population of GBM cells, cells were seeded at different concentrations of 900, 450, 225, 112, 56, 28, 14, and 7 cells per well (12 wells per condition) in 200μL of complete GBM media in ultra-low attachment 96 well plates (Corning Life Sciences). Plates were centrifuged at 300 × *g* for 5 minutes and incubated at 37 °C in 5% CO_2_ for 14 days. 25 μL of media was added every 5 days to each well. After the incubation period, spheroids were counted in each well and analysis was carried out using the online ELDA webtool (http://bioinf.wehi.edu.au/software/elda/). Spheroids were defined as homogeneous and rounded aggregates of cells measuring at least 50μm in diameter with poor cell-cell definition and a smooth surface. The percentage of wells negative for the presence of spheroids for each plating density was calculated and plotted against the number of cells seeded per well. These experiments were performed in triplicate.

### Alamar blue proliferation assay

To measure cell viability and proliferation, 3 × 10^3^ cells were seeded per well (10 wells per condition) in 200 μL of complete GBM media in a 96-well plate. Cells were kept overnight at 37 °C in 5% CO_2_. Alamar blue (USBiological Life Sciences, A1180) solution at 10% with complete GBM media and cells were incubated for 4 h. Absorbance was recorded at wavelength 570 nm and 600 nm at 4 hr, 12 hr, 24 hr, 48 h, and 72 h timepoints. Complete media without cells, including Alamar Blue, was included as a blank. These experiments were performed in triplicate.

### Cell cyte confluency assay

Cell confluency over time to measure proliferation was performed using the high-throughput live cell imaging CellCyte X (Cytena, Bico Company). Briefly, 8 × 10^3^ cells per well were seeded with GBM complete media in 12 wells per condition in a 96-well plate. Cells were allowed to attach overnight and were then placed in the CellCyte X incubator at 37 °C in 5% CO_2_ and 95% humidity for the duration of the experiment. Two random fields per well were imaged every 6 hours for a period of 5 consecutive days. Percentage of confluency data was obtained at the end of the experiment. These experiments were performed in triplicates.

### Intracranial implantation of BTICs in a murine model of GBM

The following experiments were approved by Mayo Clinic, Jacksonville FL, IACUC committee. Following rocket flight, patient-derived RF and GC BTICs (QNS108) were re-seeded into laminin-coated flasks and re-grown over two weeks for in vivo experiments. As described previously, QNS108 BTICs were implanted intracranially into 6-week-old nude mice for RF and GC cells (n = 5 per group, RF vs GC)^13,14,33^. In brief, 0.3 × 10^6^ cells were implanted with a Hamilton syringe into the right striatum (from bregma: Lateral: 1.34 mm, Anterior: 1.5 mm, Depth: 3.5 mm). Mice were monitored and sacrificed when they met end-point criteria. Murine brains were collected, fixed with 4% paraformaldehyde (PFA), paraffin-embedded, and stained with hematoxylin and eosin (H&E) and with Human Nuclei (HuNu, anti-human nuclei antibody, Millipore, MAB1281B) as previously done^13,14,33^.

### Measurement of tumor-associated cysts

Murine brains stained with H&E collected post in vivo implantation were observed to have tumor-associated cysts. All brain slices that were observed to have cystic lesions were measured for total cyst area. Murine slices with intact brains and well-defined cystic lesion were included. Brain slices that were torn during preparation were excluded from analysis. Tumor cystic areas were measured with ImageJ.

### Statistical analysis

Statistical analysis was performed using GraphPad PRISM 8.0.0 *(©2018 GraphPad Software) and R Studio (v4.2.2). Cystic tumor areas were compared with the Wilcoxon rank sum test. Survival study was analyzed with a Kaplan-Meier curve and a log rank test. All *p* values < 0.05 were considered significant.

## Data Availability

Source data is available in the supplementary section for the paper. Code used for data analysis is available at the following public GitHub repository: https://github.com/cesarga0011/npj_Brain-Tumor-Stem-Cells-Following-a-Suborbital-Rocket-Flight.
